# *Alpinia officinarum* Rhizome ameliorates the UVB induced photoaging through attenuating the phosphorylation of AKT and ERK

**DOI:** 10.1186/s12906-022-03707-w

**Published:** 2022-09-02

**Authors:** Jong Min Jung, Oh Yun Kwon, Jong Kyu Choi, Seung Ho Lee

**Affiliations:** Department of Nano-Bioengineering, Incheon National University, 119 Academy-ro, Incheon, 22012 Korea

**Keywords:** *Alpinia officinarum Rhizome*, Wrinkle formation, UVB, Epidermis, MMP-1a, COL1A1

## Abstract

**Background:**

Chronic ultraviolet (UV) exposure is one of the major external factors in skin aging, and repetitive UVB exposure induces extracellular matrix (ECM) damage as well as metabolic disease. *Alpinia officinarum* Rhizome (AOR) is a medicinal plant that has been traditionally used for treating rheumatism and whooping cough. However, the antiphotoaging effects of AOR remain unclear. We investigated the protective effects of water extracts of AOR (WEAOR) in terms of UVB-mediated ECM damage, wrinkle formation, inflammatory responses, and intracellular signaling on hairless mice and NIH-3T3 skin fibroblast cells.

**Methods:**

WEAOR was administered to UVB-irradiated hairless mice. Wrinkle formation was assessed using the replica assay, epidermal changes through H&E staining, and collagen contents in mice skin through Masson’s trichrome staining. The expression of procollagen type-1 (*COL1A1*), metalloproteinase-1a (*MMP-1a*), and inflammatory cytokines (*IL-6*, *IL-8*, and *MCP-3*) in hairless mice skin and NIH-3T3 cells was investigated through qRT-PCR. The effects of WEAOR or signaling inhibitors on UVB-induced expression of intracellular mitogen-activated protein kinases (MAPKs) were estimated by Western blotting and qRT-PCR, respectively.

**Results:**

Topical WEAOR significantly attenuated the UVB-induced wrinkle formation and epidermal thickening in the skin of hairless mice. WEAOR treatment also attenuated the UVB-induced expression of *MMP-1a* and *COL1A1* and recovered the reduction of collagen content in mouse skin. These effects were confirmed in NIH-3T3 skin fibroblast cells. WEAOR treatment restored the UVB-induced *COL1A1* and *MMP-1a* gene expression and attenuated the UVB-induced expression of *IL-6*, *IL-8*, and *MCP-3* in NIH-3T3 cells. Notably, WEAOR attenuated UVB-induced phosphorylation of AKT and ERK, but not that of p38 and JNK in NIH-3T3 cells. In addition, the administration of AKT and ERK inhibitors restored the UVB-induced expression of *MMP-1a* and *COL1A1* to an equal extent as WEAOR in NIH-3T3 cells.

**Conclusions:**

The antiphotoaging properties of WEAOR were first evaluated in this study. Our results suggest that WEAOR may be a potential antiphotoaging agent that ameliorates UVB-induced photoaging processes via the AKT and ERK signaling pathways.

**Supplementary Information:**

The online version contains supplementary material available at 10.1186/s12906-022-03707-w.

## Background

Skin aging is caused by internal factors, such as hormones and metabolites, and external factors, such as smoking and ultraviolet (UV) irradiation [1]. Repetitive exposure to UV radiation, especially high-wavelength UV radiation such as UVA (315–400 nm) and UVB (280–315 nm), is the main cause of skin aging. Photoaging induced by long-term UVB exposure is characterized by wrinkle formation [2], dyspigmentation [3], reduced elasticity, and subcutaneous fat loss [4]. Chronic UVB irradiation attenuates procollagen biosynthesis and stimulates collagen degradation, resulting in the reduction of dermal collagen contents. Therefore, many studies have focused on developing antiphotoaging agents that can regulate the expression of collagen biosynthesis–related genes, such as procollagen type-1 (*COL1A1*) and metalloproteinase-1a (*MMP-1a*) [5–7].

Increases in inflammatory cytokines (interleukin [IL]-6, IL-8, and monocyte chemotactic protein-3, [MCP-3]) have been detected in UVB-irradiated skin, which can cause acute edema and erythema [8]. In addition, UVB-mediated expression of IL-6, IL-8, and MCP-3 were reported as key regulators in UVB-induced subcutaneous fat loss [4]. Therefore, upregulation of the inflammatory cytokines stimulated by UVB is a critical step in the photoaging process in skin.

Among the various molecular cascades in UVB-irradiated skin, mitogen-activated protein kinases (MAPKs) and AKT signaling pathways are abundantly investigated because UVB-induced MAPKs and AKT signaling are closely related to the expression of collagen synthesis–related genes, such as procollagen type 1 (*COL1A1*) and metalloproteinases (*MMPs*) [6]. Therefore, attenuation of the UVB-induced activation of MAPKs and AKT pathways, especially with a natural compound, may be a useful antiphotoaging agent.

*Alpinia officinarum* belongs to the Zingiberaceae family and is widely distributed in the tropics of Asia [9]*.* It is a perennial medicinal plant containing aromatic rhizomes that have long been used as a spice in Europe [10]. *A. officinarum* rhizome (AOR) is the most medicinally active region and is widely used in China, India, and Korea for invigorating the circulatory system, treating colds, relieving stomach aches, and reducing swelling [10, 11]. AOR has hypolipidemic [12], antimicrobial [13, 14], anticancer [15–17], neuroprotective [9, 18], antioxidative [12], antidermatophytic [19], and anti-inflammatory [20–22] activities. However, few reports have addressed its effects on UVB-mediated photoaging. Because the inflammatory response is a critical step in the UVB-mediated photoaging process, we hypothesized that AOR, which has strong anti-inflammatory activity, can be used to prevent the photoaging process. In this study, we investigated the effects of water extracts of AOR (WEAOR) on UVB-irradiated NIH-3T3 mouse fibroblast cells and hairless mice and evaluated how WEAOR regulates UVB-induced photoaging.

## Methods

### Preparation of WEAOR

The WEAOR was obtained from Korea Plant Extract Bank (KPEB; https://portal.kribb.re.kr/kpeb, Daejeon, Korea), where the voucher specimen was deposited (Reference number: CW02-002) [23]. The rhizomes of *A. officinarum* (Chinese origin) were collected and authenticated by a botanist at KPEB. Next, 500 g of AOR were dried, powdered, and extracted with distilled water (1 L for 2.5 h at 100 °C). The supernatant was filtered, concentrated (DW-290, Daewoong, Choongbuk, Korea), and lyophilized using a freeze dryer (Clean-vac 12, Biotron, Kyunggi, Korea). The yield of WEAOR was 4.8% (w/w).

### Cell culture and cytotoxicity assay

NIH-3T3 cells were obtained from Korean Type Culture Collection (KTCC, Seoul, Korea) and cultured in Dulbecco’s Modified Eagle’s Medium (DMEM, HyClone, UT, USA) containing fetal bovine serum (10%, Corning, NY, USA), penicillin (100 units/mL), and streptomycin (100 μg/mL). For the cytotoxicity assay, NIH-3T3 cells (1 × 10^4^ cells/well) were seeded in a 96-well plate and cultured for 24 h at 37 °C in a CO_2_ incubator. The cell culture media was then changed to serum-free DMEM with various doses of WEAOR ranging from 0 μg/mL to 500 μg/mL and cultured for 24 h. Subsequently, 10 μL of WST-1 solution (Dozen, Seoul, Korea) was added to each well and incubated for 1 h. The absorbance was estimated at 450 nm by using the iMark Microplate Reader (Bio-Rad Laboratories, Hercules, CA, USA).

### UVB treatment

NIH-3T3 cells (2 × 10^5^/well) were seeded in a 6-well plate and incubated for 24 h at 37 °C in a CO_2_ incubator. The cell culture media was then changed to serum-free DMEM containing WEAOR or 10 µM of each inhibitor (PD98059 and LY294002) (Cell Signaling Technology, Danvers, MA, USA) and incubated for 24 h. After washing with phosphate-buffered saline (PBS), 1 mL of PBS was added to each well and irradiated with UVB (25 mJ/cm^2^) using a microprocessor-controlled UV irradiation system (BIO-LINK 312, VILBER, Suebia, Germany). The UVB-irradiated NIH-3T3 cells were further incubated with complete media (DMEM with 10% FBS, 100 units/mL of penicillin, and 100 μg/mL of streptomycin) for 24 h at 37 °C in a CO_2_ incubator and then used for the next experiments.

### Animal experiments

Six-week-old hairless mice (SKH-1, female) were obtained from Orientbio Inc. (Seoul, Korea). The mice were housed under pathogen-free conditions with a temperature of 23 ± 2 °C, humidity of 50 ± 10% and 12 h light/dark cycle. After 1 week of adaptation, the mice were divided into four groups (*n* = 5 per group): nontreated (control), UVB-irradiated (UVB), UVB-irradiated with pretreatment using WEAOR 25 μg/mL (UVB + WEAOR 25), and UVB-irradiated with pretreatment using WEAOR 50 μg/mL (UVB + WEAOR 50). WEAOR was dissolved in propylene glycol/ethanol (7:3) at a concentration of 25 μg/mL and 50 μg/mL. 100 µL of each solution were treated to the dorsal areas of the hairless mice, and UVB was irradiated using a microprocessor-controlled UV irradiation system (BIO-LINK 312, VILBER). The hairless mice were exposed to UVB from 1 MED (1 MED = 50 mJ/cm^2^) to 4 MED every other day for 10 weeks, and the total amount of UVB irradiation was 78 MED (3900 mJ/cm^2^) [23, 24]. MED is defined as the minimum dose of radiation required to make an erythema with sharp margins after 24 h.

### Skin replica assay

At the end of UVB exposure, the skin replicas were cast on the dorsal skin of the hairless mice by using a silicon-based impression material (Perfect-F Light Body Cartridge, Handae Chemical, Sungnam, Korea), and wrinkle formation was measured under a digital microscope (Nikon, Tokyo, Japan).

### Hematoxylin–eosin and Masson’s trichrome staining

Dorsal skin specimens were separated, fixed in paraformaldehyde solution (10%, w/v), dehydrated with ethanol, and then embedded in paraffin. The sliced sections (approximately 5 μm thick) were stained with hematoxylin–eosin (H&E) for estimating the epidermal changes, and Masson’s trichrome solution [7] for measuring the contents of collagen fiber in the skin tissue. The slides were photographed using a light microscope (Nikon), and the epidermal thickness and the amount of collagen fiber in the skins were estimated using ImageJ software (National Institute of Health, MD, USA).

### Quantitative realtime PCR (qRT-PCR)

The total RNA of NIH-3T3 cells and skin tissue was isolated using TRIzol Reagent (Invitrogen, Waltham, MA, USA). The ratio of UV observance at 260 nm and 280 nm (260 nm/280 nm) was measured to estimate the purity of total RNA. The ratio of total RNA used in this study was 1.8 − 2.0. Complementary DNA (cDNA) was synthesized using 1 µg of the total RNA and 10 pM of oligo dT primer, and qRT-PCR was conducted in a RT-PCR detection system (CFX Connect, Bio-Rad) using SYBR Green Realtime PCR Master Mix (Toyobo, Tokyo, Japan). The Ct method was used for estimating the relative expression of each gene and normalized to the expression of glyceraldehyde 3ʹ-phosphate dehydrogenase (*GAPDH*). The sequences of the *MCP-3* primers were 5ʹ-ATAGCCGCTGCTTTCAGCAT-3ʹ (forward) and 5ʹ-CTTCCCAGGGACACCGACTA-3ʹ (reverse). Those of the *IL-6* primers were 5ʹ-ACAACCACGGCCTTCCCT-3ʹ (forward) and 5ʹ-AGCCTCCGACTTGTGAA-3ʹ (reverse), and those of the *IL-8* primers were 5ʹ-TGTCCCATGCCACTCAGAGA-3ʹ (forward) and 5ʹ-AGCAGGTGCTCCGGTTGTAT-3ʹ (reverse). Those of *MMP-1a* were 5ʹ-ACTTTCCAGCCAGGCCCA-3ʹ (forward) and 5ʹ-CACTGCTGTTGGTCCACGT-3ʹ (reverse). Those of the procollagen type-1 (*COL1A1*) primers were 5ʹ-CACTGCTGTTGGTCCACGT-3ʹ (forward) and 5ʹ-AAAGCACAGCACTCGCCC-3ʹ (reverse). Those of the glyceraldehyde 3-phosphate dehydrogenase (*GAPDH*) primers were 5ʹ-AAGCTGTGGCGTGATGGC-3ʹ (forward) and 5ʹ-TGACCTTGCCCACAGCCT-3ʹ (reverse).

### Western blotting

NIH-3T3 cells were washed with PBS and lysed with a protein extraction buffer (20-mM Tris–HCl [pH 7.4], 150-mM NaCl, 70-μM ethylene-diamine-tetra-acetic acid, phosphatase inhibitor cocktail (Cell Signaling Technology), and Nonidet P-40 [1%, w/v]) on ice for 1 h. After centrifuging (13,000 × g, 4 °C, 15 min), the supernatants were separated and used to estimate the expression of each protein. Aliquots of the lysates (20 μg) were boiled for 10 min and electrophoresed in sodium dodecyl sulfate (SDS) polyacrylamide gel. The proteins in the SDS–polyacrylamide gel were transferred to nitrocellulose membranes and subsequently incubated with blocking buffer (5% [w/v] of nonfat milk in tris-buffered saline with Tween 20 [TBS-T]) for 1 h. After incubation with 5% nonfat milk to block the nonspecific signals, the membranes were incubated with each antibody (1:1500) for 12 h at 4 °C. Rabbit anti-phospho-AKT (4060S), rabbit anti-AKT (9272S), rabbit anti-phospho-ERK (9102S), rabbit anti-ERK (9101S), rabbit anti-phospho-JNK (9251S), rabbit anti-JNK (9252S), rabbit anti-phospho-p38 (9212S), and rabbit anti-p38 (9211S) were obtained from Cell Signaling Technology. After washing with a TBS-T buffer, the membrane was further incubated with a horseradish peroxidase (HRP)-conjugated rabbit secondary antibody (1:3000) (Santa Cruz Biotechnology, Dallas, TX, USA) at room temperature for 2 h. An enhanced chemiluminescence (ECL) detection kit (Bio-Rad) was used to visualize each protein band.

### High-performance liquid chromatography/mass spectrometry analysis

High-performance liquid chromatography (HPLC)/mass spectrometry (MS) analysis was conducted to estimate the major constituent of WEAOR. The analysis was performed on an AQUITY Ultra Performance LC system (Waters, San Jose, CA, USA) coupled with a Micromass Q-Tof Premier mass spectrometer (Waters). WEAOR was separated on an ACQUITY UPLC™ BEH C18 column (100 mm × 2.10 mm, 1.7 µm, Thermo Fisher Scientific, San Jose, CA, USA) by using a flow rate of 0.4 mL/min at 40 °C. The mobile phase of eluent A (aqueous formic acid solution, 0.1% v/v) and eluent B (acetonitrile with formic acid, 0.1%, v/v). The Micromass Q-Tof Premier MS and spray chamber conditions were capillary temperature, 350 °C; source voltage, 2.3 kV.

### Estimating sun protection factor

Determination of the sun protection factor (SPF) of WEAOR was performed according to the methods of Mansur JS et al. [25]. Briefly, WEAOR and para-amino benzoic acid were resolved in DMSO (final concentration: 1 mg/mL) respectively and UV–visible absorption spectrum was estimated using Nanodrop 2000 (Thermo Scientific). UV absorption of both solutions was estimated by spectrometer wavelengths ranging from 290 to 320 at 5-nm intervals. The Mansur equation [25] with the erythermal value provided by Sayre et al. [26] was used for estimating the SPF of WEOAR.

### Statistical analysis

All experiments were performed in triplicate and repeated three times. All data arepresented as mean ± standard deviation (SD). Two-tailed, unpaired Student’sttest and ANOVA and Tukey’s post hoc multiple comparisons using Prism 5 (Graph-Pad Software, San Diego, CA, USA) were used for statistical analysis and *P* < 0.05 was considered statistically significant.

## Results

### AOR attenuated UVB-induced wrinkle formation

**Fig. 1:**
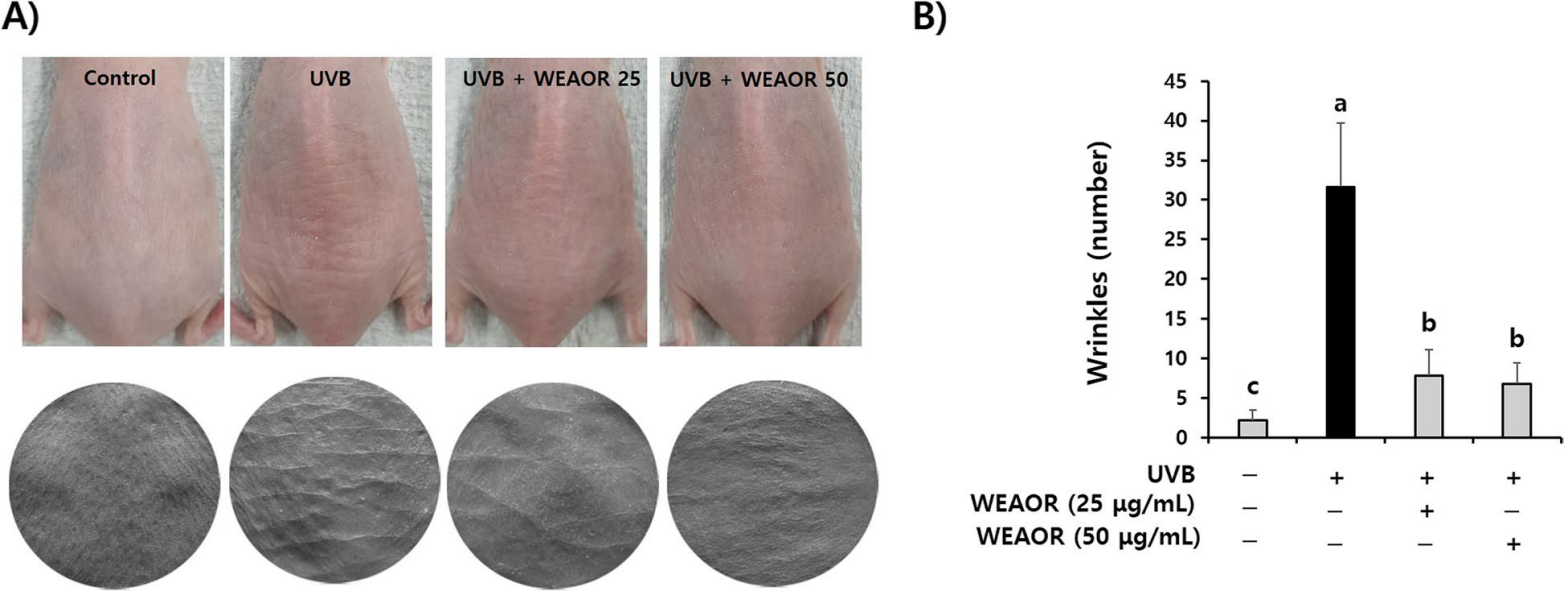
UVB-induced wrinkle formation was attenuated by water extract of *Alpinia officinarum* rhizome (WEAOR) treatment. Wrinkles were analyzed using a skin replica taken from the dorsal area of the control, UVB, UVB + WEAOR 25, and UVB + WEAOR 50 mouse groups (*n* = 5). Representative pictures of mouse dorsal area (upper panel) and skin replica (lower panel) (**A**), and the number of wrinkles was counted and tabulated (**B**). Each data value is expressed as mean ± SD. Different letters indicate significant differences between groups (*P* < 0.05)

**Fig. 2:**
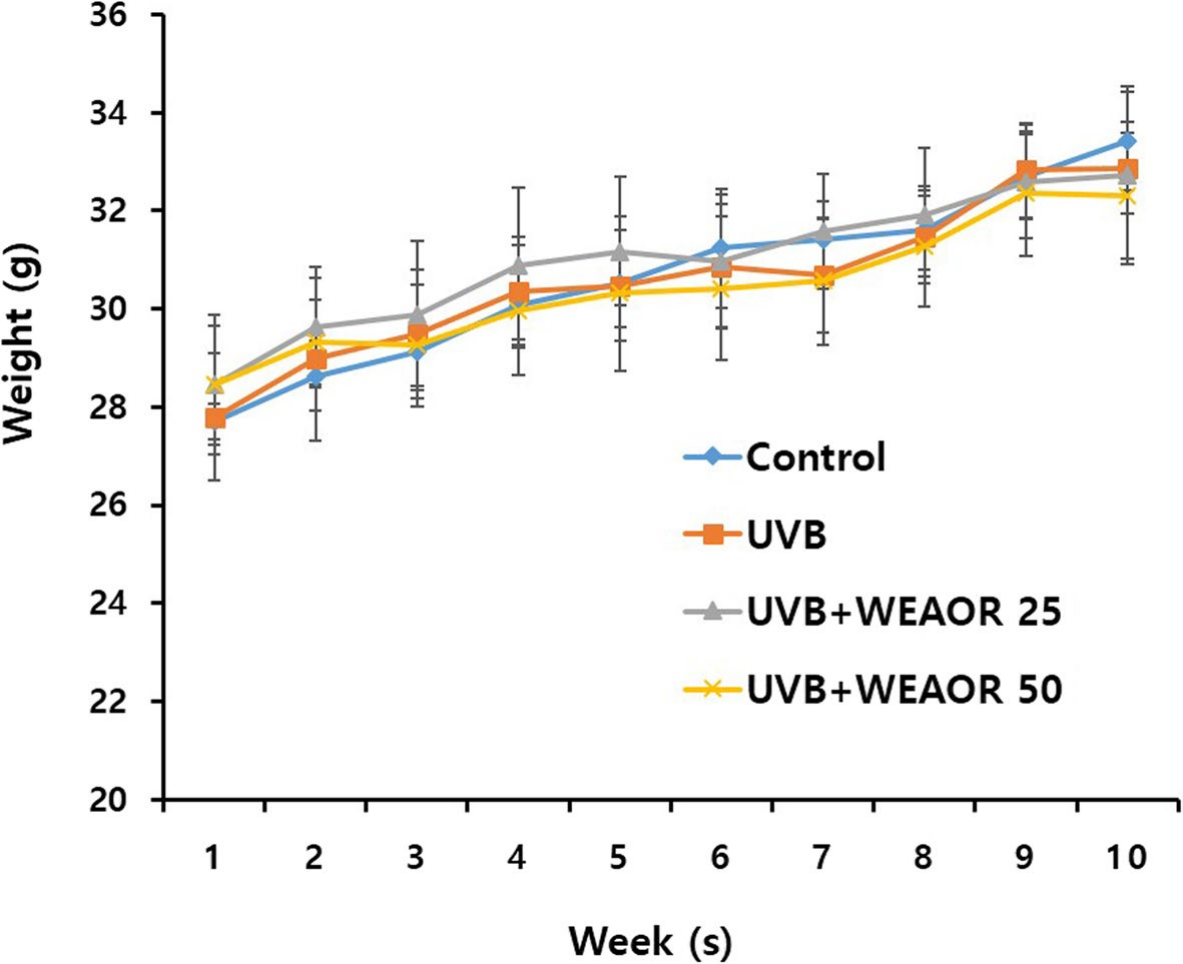
Body weight change of ultraviolet-B (UVB) irradiated mice. The body weight of each mice group (*n* = 5) was estimated once a week for 10 weeks. Each data value is expressed as mean ± SD

**Fig. 3:**
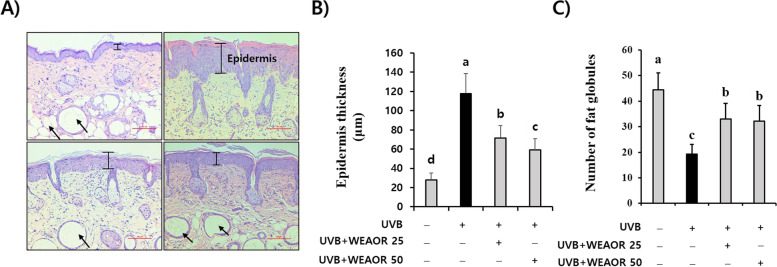
Effects of WEAOR treatment on UVB-mediated epidermal thickening and subcutaneous fat content. Administration of WEAOR inhibited UVB-induced epidermal thickening and restored UVB-induced reduction of lipid droplets in the dermis. Skin tissues from each mouse group were stained with hematoxylin& eosin (H&E) solution (**A**), and the epidermal thickening (**B**) and the number of lipid droplets (**C**) were estimated using ImageJ (*n* = 5). Each data value is expressed as mean ± SD. Scale bar 100 µm. Arrows indicate lipid droplet. Different letters indicate significant differences between groups (*P* < 0.05)

First, to estimate the antiphotoaging effects of WEAOR, we evaluated the effects of WEAOR on UVB-induced wrinkle formation using hairless mice. As illustrated in Fig. 1, the number of wrinkles on the dorsal area of the mice was dramatically increased by UVB exposure for 10 weeks (31.66 ± 7.99) but it was significantly (*P* < 0.05) decreased by WEAOR administration (WEAOR 25: 7.83 ± 3.23, WEAOR 50: 6.8 ± 2.63) without a loss of body weight (Fig. 2). In addition, we observed that UVB-induced epidermal thickness (117.75 ± 20.87 µm) was dramatically increased compared with that of the control (27.95 ± 7.44 µm). However, it was significantly (*P* < 0.05) decreased in WEAOR administered mouse skins (WEAOR 25: 71.60 ± 12.75 µm, WEAOR 50: 59.17 ± 11.82 µm) (Fig. 3A and B). Notably, the number of lipid droplets on the subcutaneous area was significantly (*P* < 0.05) reduced in the UVB-irradiated mouse skin (19.2 ± 3.83) compared with that of the control (44.4 ± 6.58) but it was significantly (*P* < 0.05) recovered by WEAOR administration (WEAOR 25: 33 ± 6.20, WEAOR 50: 32.2 ± 6.01) (Fig. 3A and C). These data suggest that the administration of WEAOR to the dorsal skin of UVB-irradiated mice can suppress UVB-induced epidermal thickening as well as UVB-induced reduction of subcutaneous fat.

### UVB-induced collagen reduction was recovered by WEAOR administration

**Fig. 4:**
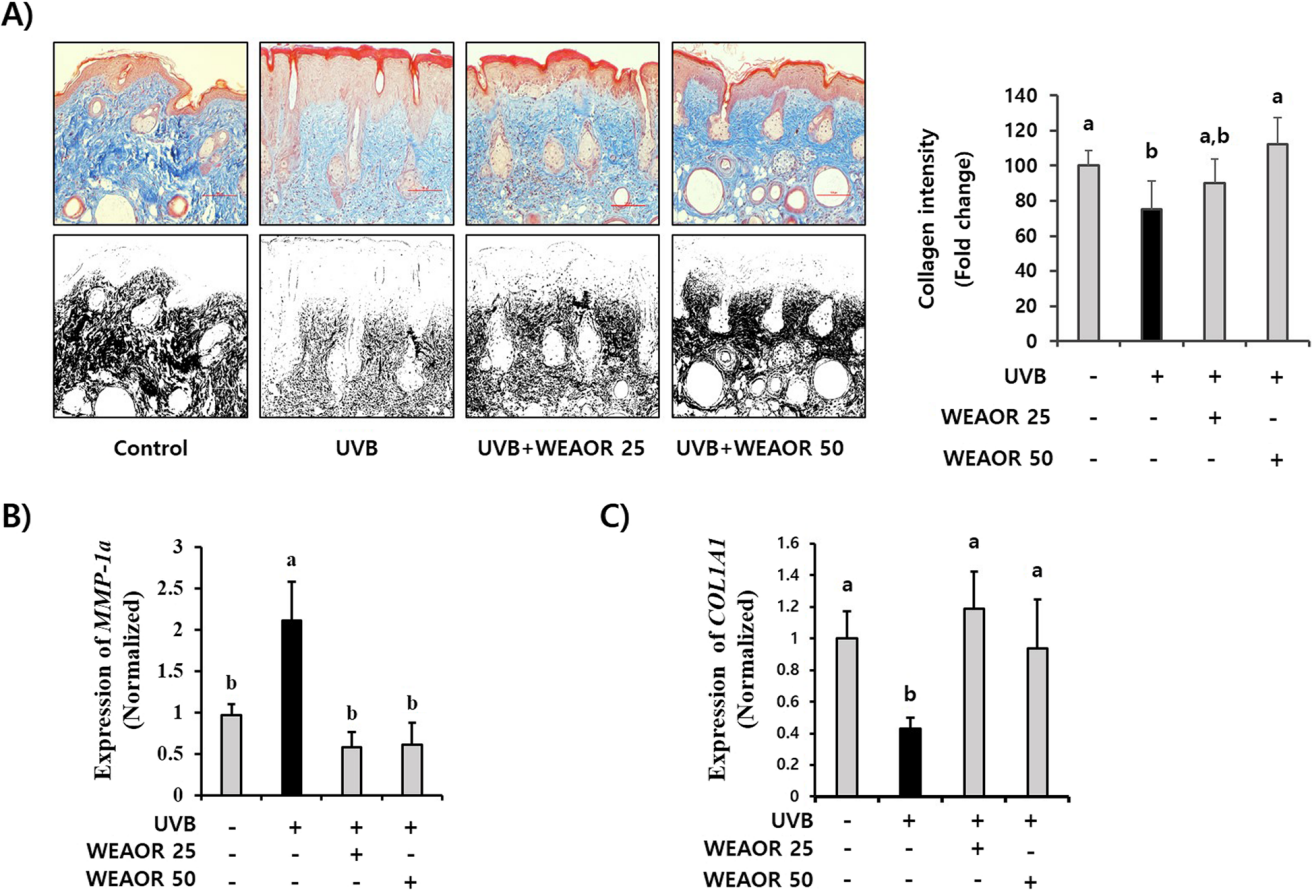
Protective effects of WEAOR on the UVB-mediated loss of collagen fibers in vivo. The collagen fiber content was estimated using Masson’s trichrome staining (*n* = 5) (**A**). All colors except blue (collagen fibers) in pictures of Masson’s trichrome staining were eliminated using Adobe Photoshop, and the blue color was desaturated (lower panel of A). The intensity of the desaturated area in each slide was measured using ImageJ and tabulated. The total RNA was isolated from the dorsal skin of each mouse group, and the effects of WEAOR on the UVB-induced expression of procollagen type-1 (*COL1A1*) (**B**) and metalloproteinase-1a (*MMP-1a*) (**C**) were evaluated using qRT-PCR. Administration of WEAOR to UVB-irradiated mouse skin recovered the loss of collagen and the abnormal expression of *COL1A1* and *MMP-1a*. Each data value is expressed as mean ± SD. Scale bar 100 µm. Different letters indicate significant differences between groups (*P* < 0.05)

We investigated the effects of WEAOR on UVB-induced skin collagen content changes that play a vital role in maintaining skin elasticity. As illustrated in Fig. 4A, the UVB-treated skin collagen content (75.02 ± 16.37), which was estimated by Masson’s trichrome staining, was reduced compared with that of the control (100 ± 8.70). However, the density of the trichrome positive area (blue) (112.44 ± 15.18) in the WEAOR (50 µg/mL)-treated skin tissue was significantly (*P* < 0.05) increased compared with that of the UV-treated animal group. In addition, UVB-induced expression of *COL1A1* (2.11 ± 0.46) and *MMP-1a* (0.87 ± 0.14) in the mouse skin was significantly (*P* < 0.05) recovered in the WEAOR-treated animal groups (*MMP-1a* of WEAOR 25: 0.577 ± 0.18, WEAOR 50: 0.60 ± 0.26; *COL1A1* of WEAOR 25: 2.429 ± 0.48, WEAOR 50: 1.92 ± 0.63) (Fig. 4B and C). These data suggest that the administration of WEAOR to the mouse dorsal skin area inhibited UVB-mediated collagen reduction by regulating the expression of *COL1A1* and *MMP-1a*.

### WEAOR attenuated the expression of photoaging-related genes in NIH-3T3 cells

**Fig. 5:**
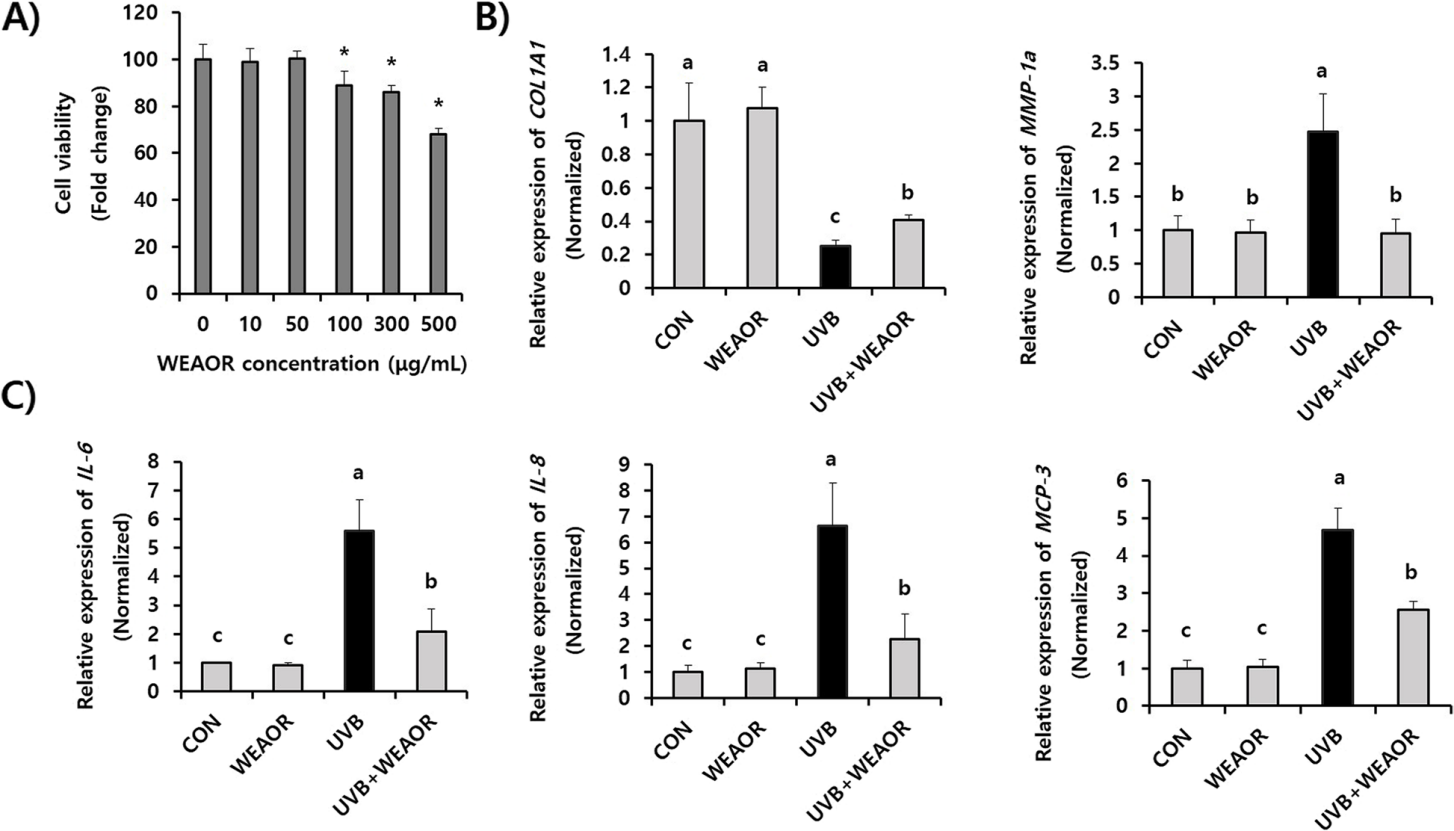
Administration of nontoxicological levels of WEAOR attenuated the expression of photoaging-related genes. Cytotoxicity of WEAOR on NIH-3T3 cells (**A**). NIH-3T3 cells were treated with various concentrations of WEAOR (0–500 μg) and incubated for 24 h at 37 °C in a CO_2_ incubator. UVB-mediated expression of *COL1A1* and *MMP-1a* (**B**), and *IL-6*, *IL-8*, and MCP-3 (**C**) were significantly restored by WEAOR treatment. The relative expression of each gene was estimated using qRT-PCR and normalized by that of *GAPDH*. Experiments were conducted in triplicate and repeated three times with similar results. Each data value is expressed as mean ± SD. * indicates a significant difference compared with the control (nontreated). Different letters indicate significant differences between groups (*P* < 0.05)

We estimated the effects of WEAOR on UVB-irradiated NIH-3T3 skin fibroblast cells. To determine the cytotoxic levels of WEAOR on NIH-3T3 cells, various concentrations of WEAOR were administered to NIH-3T3 cells, and the cell viability was investigated using a wst-1 cell viability assay kit. As illustrated in Fig. 5A, the administration of WEAOR up to a concentration of 50 µg/mL did not show cytotoxicity on NIH-3T3 cells. On the basis of these results, we then investigated the effects of nontoxicological levels of WEAOR (50 µg/mL) on the UVB-induced expression of photoaging-related genes, such as *COL1A1* and metalloproteinase-1a (*MMP-1a*). UVB irradiation on the NIH-3T3 cells reduced the expression of *COL1A1* (0.25 ± 0.03), which is important in collagen synthesis, and increased the expression levels of *MMP-1a* (2.47 ± 0.57), which can degrade collagen in skin, compared with that of the control (*COL1A1*: 1 ± 0.22, *MMP-1a*: 1 ± 0.21). However, the administration of WEAOR (50 µg/mL) significantly restored the UVB-mediated reduction of *COL1A1* (0.40 ± 0.03) and increase of *MMP-1a* (0.95 ± 0.21) in NIH-3T3 cells (Fig. 5B). Furthermore, we observed that UVB-induced expression of proinflammatory cytokines, such as *IL-6* (5.58 ± 1.09), *IL-8* (6.62 ± 1.66) and *MCP-3* (4.69 ± 0.67), which are the main causes of UVB-induced skin inflammation, was significantly (*P* < 0.05) attenuated by WEAOR (*IL-6*: 2.07 ± 0.89, *IL-8*: 2.26 ± 0.95, and *MCP-3*: 2.56 ± 0.68) (Fig. 5C). These results suggest that WEAOR can be used to attenuate photoaging in terms of reduced collagen degradation and skin inflammation.

### ERK and AKT intracellular signaling are key regulators of the WEAOR-mediated antiphotoaging process

**Fig. 6:**
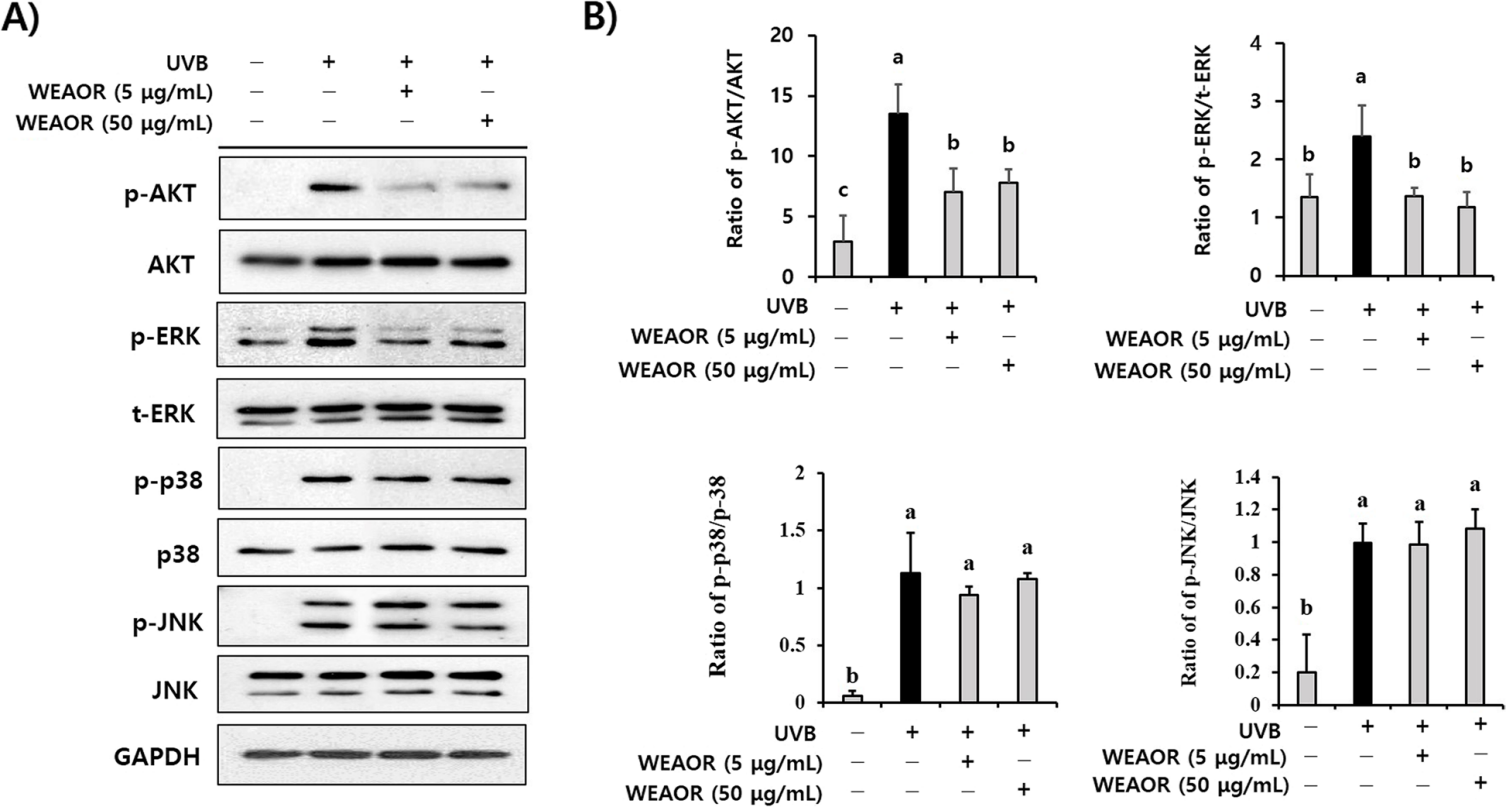
ERK and AKT signaling are key regulators in WEAOR-mediated antiphotoaging activity. Pretreatment of WEAOR to NIH-3T3 cells attenuated phosphorylation of ERK and AKT, but not that of JNK and p38 MAPK. Representative figures of Western blotting are shown (**A**), and the intensity of each band was estimated using ImageJ. The relative expression rates of ERK (p-ERK/t-ERK), p38 (p-p38/p38), JNK (p-JNK/JNK), and AKT (p-AKT/AKT) were calculated and tabulated (**B**). Experiments were conducted in triplicate and repeated three times with similar results. Each data value is expressed as mean ± SD. Different letters indicate significant differences between groups (*P* < 0.05)

**Fig. 7:**
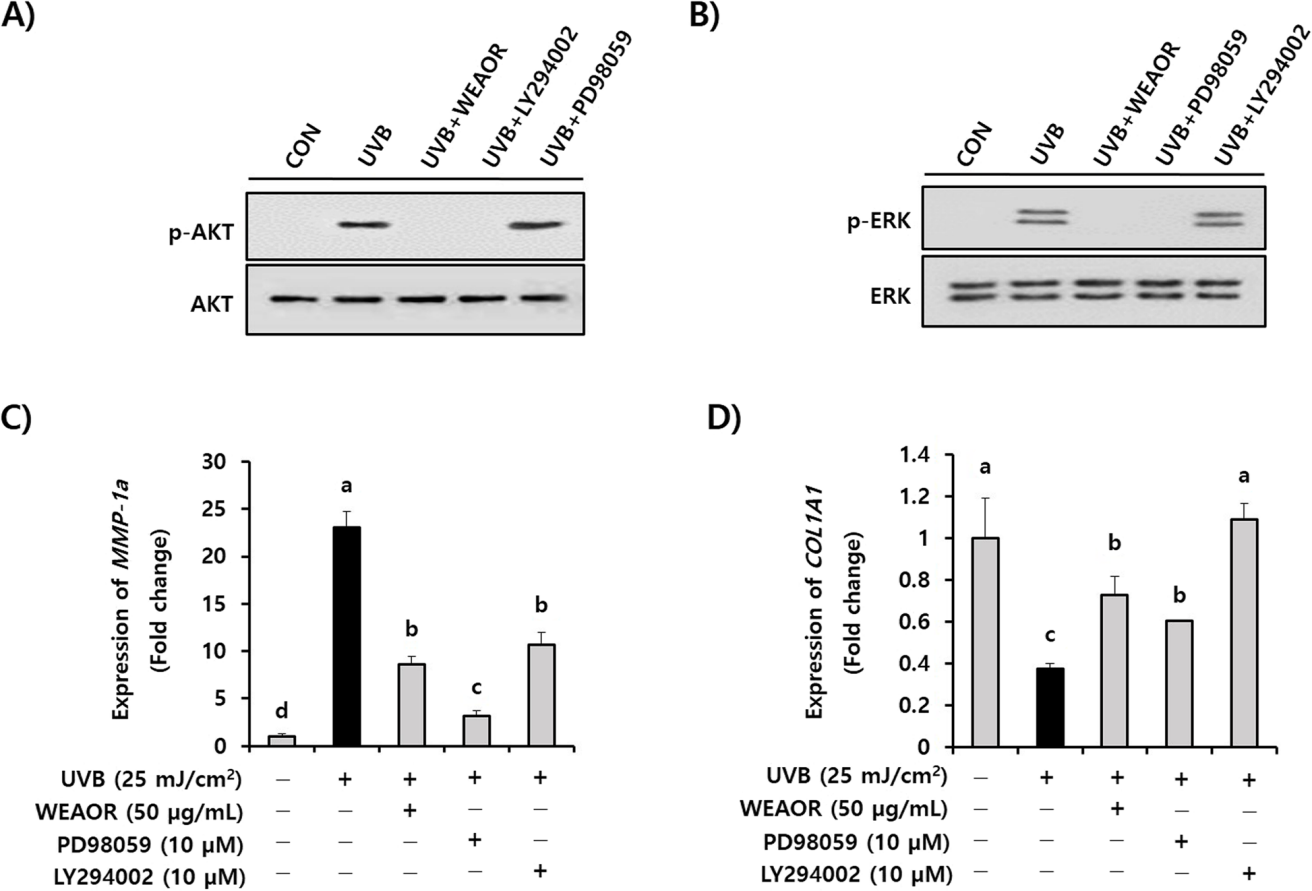
UVB-induced reduction of *COL1A1* and increase of *MMP-1a* were restored by administration of WEAOR as well as by signaling inhibitors (PD98059: MEK inhibitor and LY294002: AKT inhibitor). The effects of WEAOR, PD98059, LY294002 on the UVB-induced activation of ERK and AKT was estimated by western blotting (**A**, **B**). The UVB-mediated expression of *COL1A1* and *MMP-1a* on NIH-3T3 cells was estimated by qRT-PCR (**C**, **D**). Experiments were conducted in triplicate and repeated three times with similar results. Each data value shown is mean ± SD. Different letters show significant differences between groups (*P* < 0.05)

To identify the key regulators of the WEAOR-mediated antiphotoaging process, the effects of WEAOR on MAPKs and AKT phosphorylation were investigated in NIH-3T3 cells. UVB irradiation phosphorylated intracellular MAPKs and AKT molecules. However, the administration of WEAOR to NIH-3T3 cells significantly (*P* < 0.05) attenuated the UVB-induced phosphorylation of ERK and AKT but not that of JNK and p38 MAPK (Fig. 6). Furthermore, after confirming the effects of PD98059 and LY294002 on the activation of ERK and AKT (Fig. 7A and B), we observed that treatment with PD98059 (MEK inhibitor, 10 µmol) and LY294002 (AKT inhibitor, 10 µmol) significantly (*P* < 0.05) recovered the UVB-induced *COL1A1* (0.42 ± 0.08) and *MMP-1a* (23.08 ± 1.67) to the same extent as WEAOR treatment (50 µg/mL) in NIH-3T3 cells (*COL1A1* of WEAOH treatment: 0.64 ± 0.16, PD98059 treatment: 0.57 ± 0.05, LY294002 treatment: 1.161 ± 0.13; *MMP-1a* of WEAOH treatment: 8.57 ± 0.88, PD98059 treatment: 3.132 ± 0.59, LY294002 treatment: 10.68 ± 1.25) (Fig. 7C and D). Taken together, these data strongly suggest that WEAOR attenuates the UVB-induced photoaging process by regulating the intracellular phosphorylation of AKT and ERK.

### Analysis of WEAOR

**Fig. 8:**
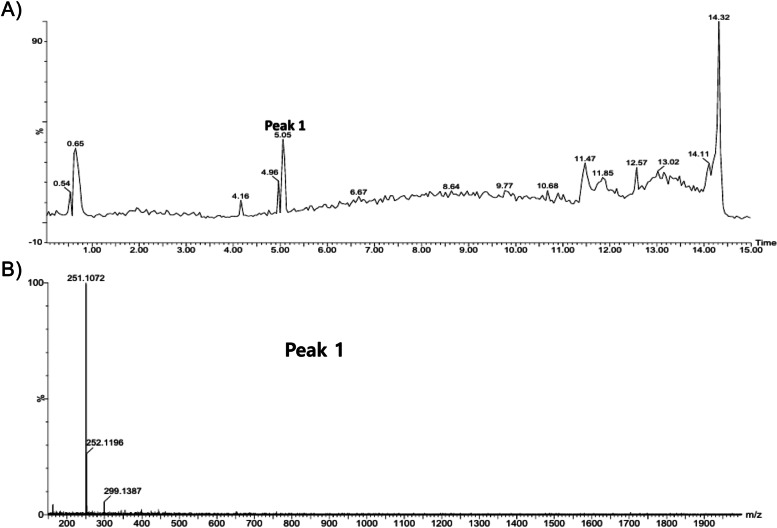
HPLC–MS analysis of WEAOR. Peak 1 (tR 5.05) of the HPLC chromatogram of WEAOR (**A**) was selected for further analysis by a mass spectrometer (**B**)

WEAOR was analyzed with HPLC/MASS to validate the single constituent that can be used as an analytical indicator in making WEAOR. WEAOR was subjected to HPLC (Fig. 8A), and a major peak (t_R_ 5.05) was further analyzed with a mass spectrometer (Fig. 8B). The observed mass of a major peak is presented in Table 1. By referencing the reported results, peak 1, which is a major constituent of WEAOR, may correspond to kaempferide.

**Table 1:** Major constituent of WEAOR

Natural product	Peak	RT (min)	Observed mass	Fragment ion	Single compound	Formula	Molecular mass (g/mol)	Ref
*Alpinia officinarum r*hizome	Peak 1	5.05	299.1387	251.1072	Kaempferide	C**16**H**12**O6	300.26	[27]

### Sunscreen effects of WEAOR

The sunscreen effect of natural materials is essential for antiphotoaging material development. Several phytochemicals such as caffeic acid [28] and naringin [29], isolated from natural resources, have been reported to have sunscreen properties. Notably, we observed that the SPF value of WEAOR was 3.43, which is a remarkable value compared with that of para-aminobenzoic acid (13.86). In addition, WEAOR showed maximum UV absorbance at 315 nm, with a range of UVB (290–320 nm) (Table 2). These results suggest that the sunscreen property of WEAOR may affect WEAOR-mediated antiphotoaging events.

**Table 2:** Sun protection factor (SPF) of WEAOR

Wavelength, nm	EE x *I*	Absorbance for WEAOR (Abs1)	Absorbance for para-amino benzoic acid (Abs2)	Abs1 x EE x *I* (WEAOR)	Abs2 x EE x *I* (para-amino benzoic acid)
290	0.0150	0.104	2.272	0.00157	0.03408
295	0.0817	0.083	1.987	0.00678	0.16192
300	0.2874	0.073	1.842	0.02117	0.52958
305	0.3278	0.287	1.317	0.09407	0.43171
310	0.1864	0.671	0.820	0.12513	0.15291
315	0.0837	1.124	0.804	0.09407	0.06735
320	0.0180	0.023	0.509	0.00042	0.00916
Total				0.34323	1.38673
Total x CF (10)				3.43236	13.86738
SPF values				3.43	13.86

*EE* The erythemal effect spectrum, *I* The solar intensity spectrum, *Abs* The absorbance of sunscreen product, *CF* The correction factor (= 10)

## Discussion

The destruction of the ozone layer due to environmental pollution has increased the amount of harmful ultraviolet (UV) radiation reaching the Earth, resulting in an increase in the number of patients with skin aging and skin diseases. Although many chemical-based materials, such as benzophenone and its derivates, have been developed to absorb UV rays [30], the demand for less toxic and long-term usable antiphotoaging agents continues to grow [27].

Skin aging by UV rays is characterized by wrinkle formation, epidermal thickening, and reduced collagen content in the dermis layer. The most abundant protein in the dermis is collagen. It is a primary structural component of the dermis and is responsible for skin strength and support [19]. Therefore, inhibiting the UV-mediated loss of collagen can effectively prevent photoaging. In our study, WEAOR inhibited the UVB-mediated expression of *MMP-1a* and *COL1A1* genes, which are closely related to maintaining the collagen contents of mouse skin. Moreover, UVB-mediated loss of epidermal collagen, estimated using Masson’s trichrome staining, recovered with WEAOR treatments. Taken together, these results suggest that the natural agent WEAOR can help inhibit UVB-mediated collagen degradation.

Chronic exposure to UVB can induce inflammatory responses in the skin. Because skin plays a vital role as a barrier against foreign invaders, if the skin is excessively exposed to UVB, inflammatory responses will increase, weaken the skin’s immune system, and eventually cause the skin’s barrier function to deteriorate. Therefore, the inhibition of the UVB-mediated expression of inflammatory cytokines is a very important issue for maintaining skin health by promoting skin immunity. In addition, inhibiting the UVB-induced expression of inflammatory cytokines such as *IL-6*, *IL-8*, and *MCP-3* by their specific inhibitors may recover the UVB-mediated reduction of lipid synthesis–related genes in skin fibroblast cells [31], suggesting that ameliorating the UVB-induced expression of inflammatory cytokines can effectively prevent subcutaneous fat loss, a process of photoaging. Topical application of *Nelumbo nucifera* leaf extracts attenuated the UVB-induced subcutaneous fat loss and photoaging by inhibiting the UVB-mediated expression of inflammatory cytokines [32]. On the basis of these reports, we assessed whether WEAOR suppresses UVB-mediated expression of inflammatory cytokines. We observed that the administration of WEAOR to skin fibroblast cells effectively prevented UVB-induced expression of inflammatory cytokines such as *IL-6*, *IL-8*, and *MCP-3*. Furthermore, UVB-mediated reduction of lipid droplets in hairless mouse skin was significantly recovered in WEAOR-treated mouse skin, which is thought to be a result of the attenuation of inflammatory cytokines by WEAOR treatment. Taken together, our data suggest that WEAOR can inhibit the UVB-induced expression of inflammatory cytokines, thereby maintaining skin immunity and inhibiting the UVB-mediated reduction of subcutaneous fat.

MAPKs and AKT pathways are well-known intracellular signaling pathways involved in regulating the gene expression induced by UVB in skin tissue. For example, UVB-induced expression of *COL1A1* was closely related to MAPKs and PI3K/AKT pathways [33, 34] and the expression of IL-6 and IL-8 stimulated by UVB in HaCat human keratinocyte cells was also associated with MAPKs [35]. Kim et al. [8] reported that oral administration of the p38 MAPK inhibitor attenuated the UVB-induced expression of IL-6 and IL-8 in skin tissue. These reports suggest that inhibiting UVB-induced MAPK activation can prevent photoaging. Our data indicate that pretreatment of WEAOR to NIH-3T3 skin fibroblast cells significantly attenuated the UVB-induced phosphorylation of AKT and ERK but not that of p38 MAPK and JNK, indicating that WEAOR may attenuate photoaging processes through inhibiting AKT- and ERK-specific signaling pathways. Furthermore, WEAOR (50 μg/mL) has equivalent activity to AKT- and ERK-specific inhibitors (10 μM) in recovering the abnormal expression of *MMP-1a* and *COL1A1* by UVB. These results strongly suggest that WEAOR could be developed as a potential antiphotoaging agent to regulate UVB-induced intracellular signaling pathways such as those of AKT and ERK.

AOR has been used as a flavoring agent and as a spice [18]. Because of its pharmaceutical properties, AOR has been used as a traditional medicine to cure rheumatism and whooping cough [36]. It has anticancer, antimicrobial, and antioxidant activities [37], but few studies have evaluatedits antiphotoaging effects. Although we could not determine the single functional component of WEAOR, kaempferide was determined as a major component of WEAOR by HPLC–MS analysis (Fig. 8 and Table 1), and we think that it could be used as an analytical indicator in making WEAOR. Kaempferide is a constituent of methanol extract of AOR and may have strong anti-inflammatory properties [38]. Therefore, it may have antiphotoaging activity. We plan to perform a functional study of kaempferide in the UVB-induced photoaging process to elucidate its antiphotoaging properties.

This study has some limitations. We estimated the effects of WEAOR were estimated at the gene levels; estimating the effects of WEAOR on photoaging-related molecules at the protein level may have provided a stronger evidence of its antiphotoaging effects. Nevertheless, we clearly demonstrated the effects of WEAOR on the expression of *COL1A1* and *MMP-1a* genes on UVB-irradiated hairless mouse skin (in vivo model) and further confirmed this on UVB-irradiated NIH-3T3 skin fibroblast cells (in vitro model). Therefore, our data provide strong evidence for the antiphotoaging properties of WEAOR. Future studies should evaluate the effects of WEAOR on photoaging-related genes in protein levels.

## Conclusion

In this study, the antiphotoaging activity of *AOR* was first evaluated using UVB-irradiated NIH-3T3 skin fibroblasts and SKH-1 hairless mice. We observed that the application of WEAOR to the dorsal area of SKH-1 hairless mice effectively inhibited the photoaging processes such as wrinkle formation, epidermal thickening, and loss of dermal collagen contents. Moreover, the administration of WEAOR to UVB-irradiated NIH-3T3 cells attenuated the expression of collagen synthesis–related genes such as *COL1A1* and MMP-1a and the expression of proinflammatory cytokines such as IL-6, IL-8, and MCP-3. Finally, the involvement of the ERK and AKT signaling pathways in WEAOR-mediated antiphotoaging activity suggests that WEAOR has strong antiphotoaging activity by regulating UVB-induced ERK and AKT signaling.

## Supplementary Information

**Additional file 1.**

## Data Availability

The data used to support the findings of this study are available from the corresponding author upon request.
