# Supplementary figures and images for: Alpinia officinarum Rhizome ameliorates the UVB induced photoaging through attenuating the phosphorylation of AKT and ERK
Source: BMC Complement Med Ther. 2022 Sep 2;22:232. doi: 10.1186/s12906-022-03707-w (PMC9487146; doi:10.1186/s12906-022-03707-w)

Figure 6A.

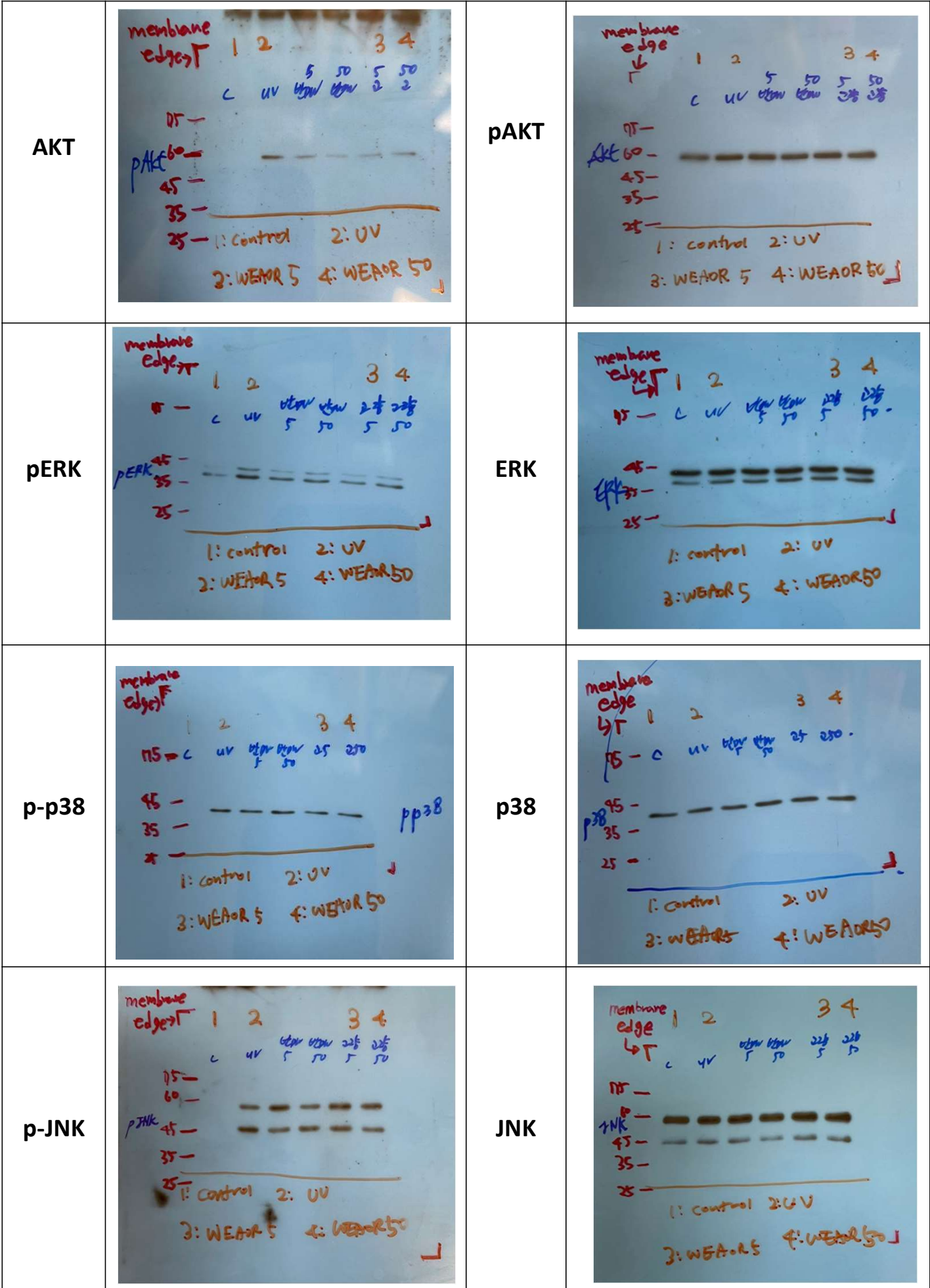

Figure 6A.

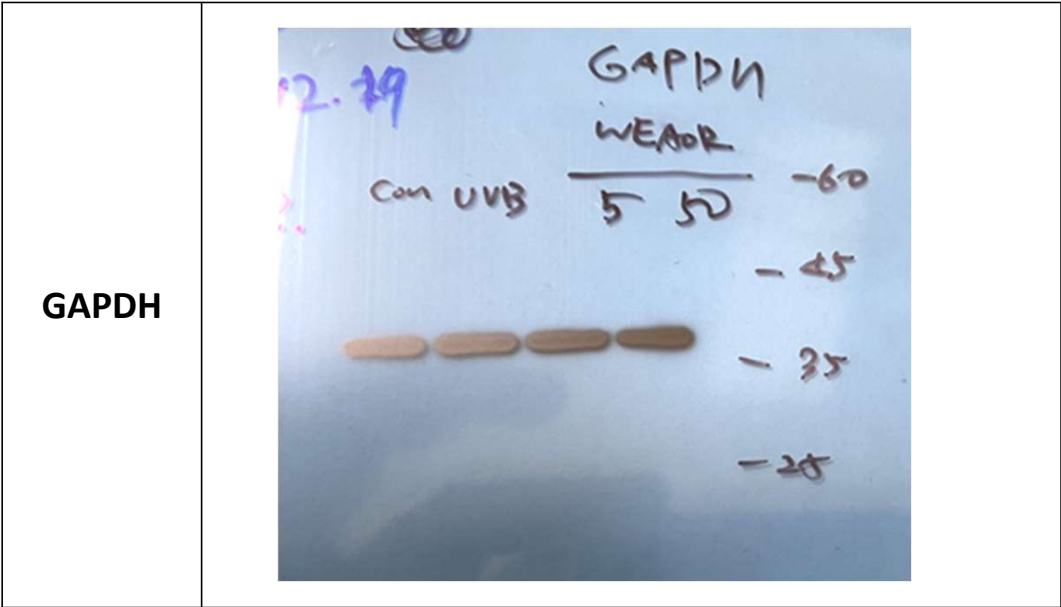

Figure 7A, B

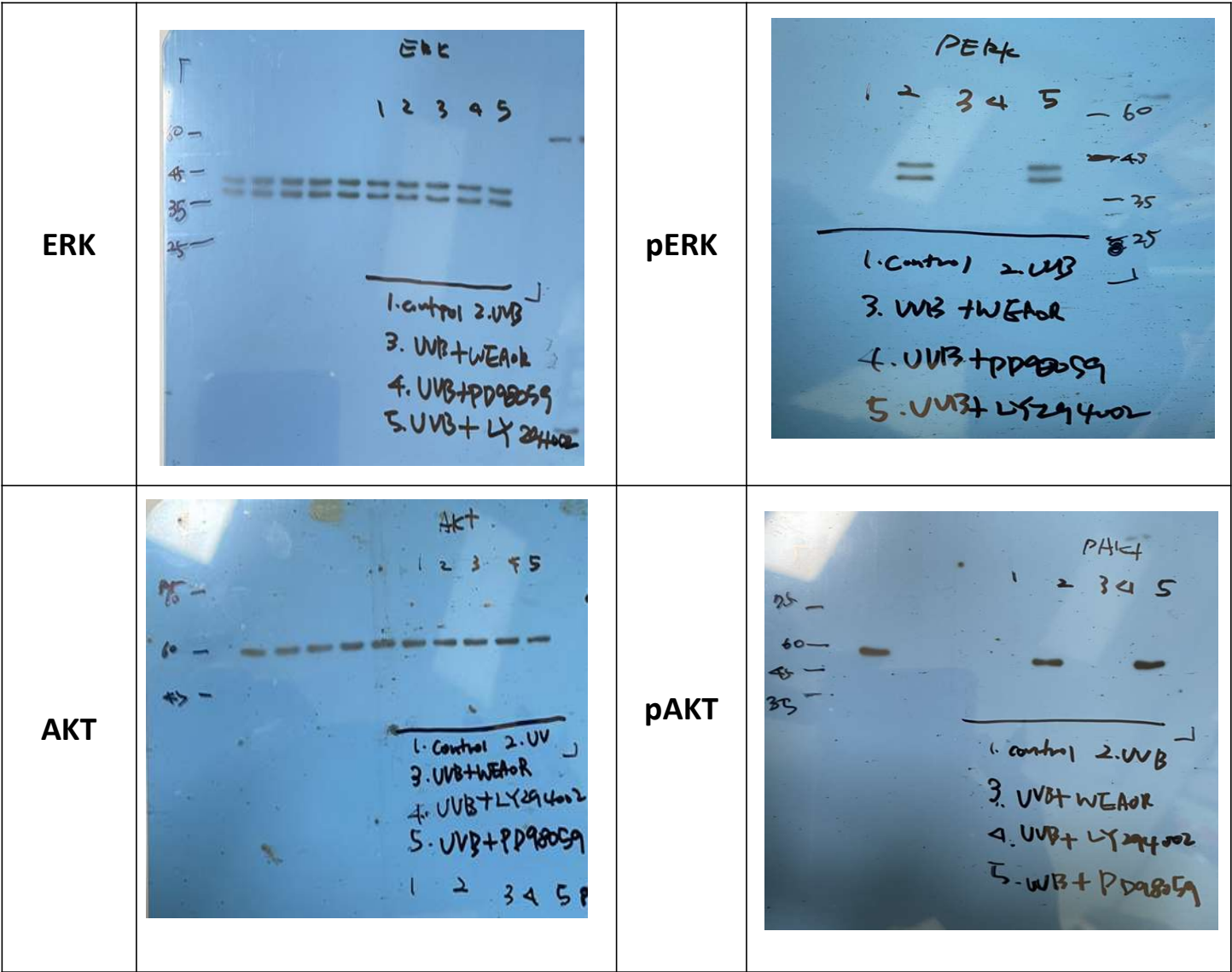

Supplement: Supplementary file 1 — Additional file 1. [file 12906_2022_3707_MOESM1_ESM.pdf]
